# Species Combinations Shape Vegetation and Soil Nutrient Responses to Grass–Legume Reseeding in a Degraded Semi-Arid Grassland

**DOI:** 10.3390/plants15203228

**Published:** 2026-10-21

**Authors:** You Xu, Guang Liu, Tian-Sheng Fu, Gui-Hong Fu, Jin-Xin Zhang, Chui-Hua Kong

**Affiliations:** 1 Institute of Ecological Conservation and Restoration, Chinese Academy of Forestry, Beijing 100091, China; xuyou406@126.com (Y.X.); 15909124583@163.com (G.L.); 2 Zhalute Banner Caoyuan Agriculture and Animal Husbandry Investment and Development Group Co., Ltd., Tongliao 029100, China; tjjfts@163.com; 3 Zhalute Banner Lubei Town Comprehensive Support and Technology Extension Center, Tongliao 029100, China; lbz2056@126.com; 4 College of Resources and Environmental Sciences, China Agricultural University, Beijing 100193, China

**Keywords:** degraded grassland, grass–legume mixture, plant diversity, reseeding, soil nutrients

## Abstract

Reseeding is widely used to restore degraded grasslands, but the extent to which restoration outcomes depend on grass–legume species combinations remains unclear. We conducted a two-year field experiment in a degraded semi-arid grassland in Inner Mongolia, China, using two grasses (*Elytrigia elongata* and *Bromus inermis*) and two legumes (*Caragana microphylla* and *Lespedeza davurica*) reseeded as monocultures or grass–legume mixtures. Reseeding resulted in higher vegetation cover than the unseeded control across all treatments, whereas biomass responses depended on species combination, with *B. inermis* + *L. davurica* showing the highest mean biomass and cover in both years. Most reseeding treatments reduced Shannon–Wiener and Simpson diversity, whereas *E. elongata* + *C. microphylla* maintained both indices at levels comparable to the unseeded control. Legume-containing treatments consistently increased soil total N, ammonium nitrogen, nitrate N, and microbial biomass N, while available P and microbial biomass C increased across all reseeding treatments. Vegetation productivity was positively associated with soil nutrient availability and microbial biomass but negatively associated with plant diversity. Overall, reseeding promoted early vegetation and soil recovery, but grass–legume mixtures were not universally superior to monocultures. These findings suggest that *B. inermis* + *L. davurica* may be preferred for rapid vegetation recovery, whereas *E. elongata* + *C. microphylla* may be considered when maintaining plant diversity is a priority.

## 1. Introduction

Grassland degradation is a widespread ecological challenge in arid and semi-arid regions, where prolonged grazing and soil erosion can reduce vegetation cover and productivity and impair soil functioning [1,2]. Grazing exclusion can promote natural recovery, but its effectiveness varies with environmental conditions and degradation severity [3,4]. In severely degraded grasslands, active restoration may therefore be needed to overcome constraints on natural recovery [5]. Reseeding is widely used for this purpose and generally increases vegetation cover and aboveground biomass and improves soil properties [6,7]. However, its effects vary with grassland type, degradation level, restoration duration, and the species used [6,8], highlighting species selection as a key determinant of restoration success.

Species composition can influence restoration outcomes through differences in resource acquisition and ecosystem effects. Mixtures of species with contrasting functional traits may use resources more efficiently than monocultures through niche complementarity and facilitation [9,10]. Grass–legume mixtures are particularly promising because legumes can acquire nitrogen (N) through symbiotic N_2_ fixation, whereas grasses rely primarily on soil mineral N, potentially reducing competition for N and promoting complementary resource use [11,12]. Such complementarity can enhance biomass production and N use [9,13], while legume-derived litter and root inputs may also influence soil nutrient cycling and fertility [14]. However, the magnitude of these effects can depend strongly on the identities and interactions of the species involved.

Restoration responses may also differ among ecosystem attributes. Although reseeding often increases vegetation cover and biomass, its effects on plant diversity are less consistent and can vary with restoration method, environmental context, and restoration duration [6,15]. Moreover, mixture performance can vary markedly among species combinations even when functional-group composition is similar, indicating an important role of species identity [16,17]. Previous reseeding meta-analyses have mainly evaluated average restoration responses across broad environmental contexts [6,7,8], whereas field studies of grass–legume mixtures have shown that responses can vary markedly with species identity and composition [18,19]. By simultaneously assessing vegetation productivity, plant diversity, soil nutrients, and microbial biomass across multiple species combinations, our study evaluates whether particular combinations provide consistent benefits or involve trade-offs among restoration objectives.

Here, we conducted a two-year field experiment in a degraded semi-arid grassland on the northwestern fringe of the Horqin Sandy Land, Inner Mongolia, China. Two grasses (*Elytrigia elongata* and *Bromus inermis*) and two legumes (*Caragana microphylla* and *Lespedeza davurica*) were reseeded as monocultures or in all pairwise grass–legume combinations. We evaluated their effects on vegetation productivity and cover, plant diversity, soil nutrients, and microbial biomass. We hypothesized that (1) reseeding would enhance vegetation recovery, soil nutrient availability, and microbial biomass relative to the unseeded control; and (2) the effects of grass–legume mixtures on vegetation productivity and soil N availability would depend on species combination, with some mixtures expected to outperform monocultures. We further examined whether different combinations produced consistent or contrasting responses across vegetation, diversity, and soil attributes. Together, this study aimed to clarify how species identity and species combination influence multidimensional restoration outcomes in degraded semi-arid grasslands.

## 2. Results

### 2.1. Effects of Reseeding Treatments on Aboveground Biomass and Vegetation Cover

**Figure 1:**
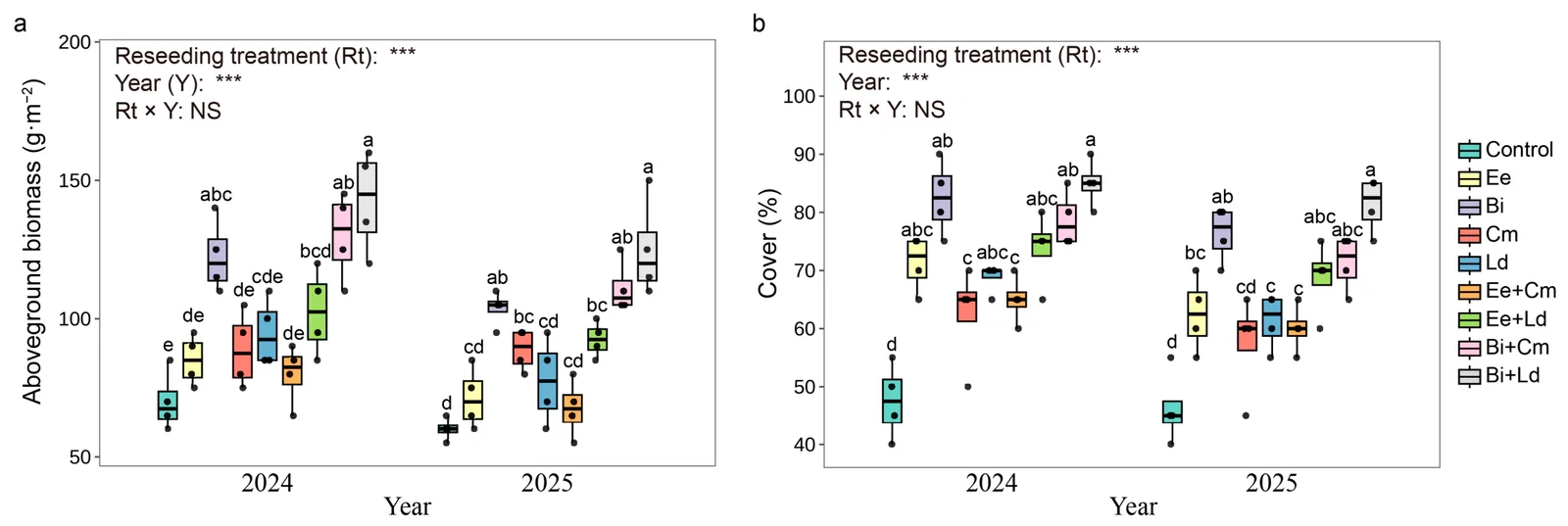
Effects of different reseeding treatments on (**a**) aboveground biomass and (**b**) vegetation cover in 2024 and 2025. Ee (*E. elongata*), Bi (*B. inermis*), Cm (*C. microphylla*), and Ld (*L. davurica*) represent four monocultures; Ee + Cm, Ee + Ld, Bi + Cm, and Bi + Ld represent four grass–legume mixtures. Data are presented as box plots with individual data points representing four independent experimental plots per treatment (*n* = 4). Boxes indicate the interquartile range, horizontal lines represent the median, and whiskers extend to the most extreme observations within 1.5 times the interquartile range. Different letters indicate significant differences among reseeding treatments within each year according to Tukey’s HSD test (*p* < 0.05). Treatments sharing at least one letter are not significantly different, whereas treatments with no letters in common differ significantly. Results of linear mixed-effects models for the effects of reseeding treatment (Rt), year (Y), and their interaction (Rt × Y) on aboveground biomass and vegetation cover are shown. ***: *p* < 0.001; NS: *p* > 0.05.

Reseeding treatment and year significantly affected aboveground biomass, whereas their interaction was not significant (Figure 1a; Table S1). In 2024, aboveground biomass was significantly higher in the Bi (*B. inermis*) and Bi + Ld (*L. davurica*) treatments than in the control. In 2025, significantly higher aboveground biomass than in the control was observed in the Bi, Cm (*C. microphylla*), Ee (*E. elongata*) + Ld, Bi + Cm, and Bi + Ld treatments. Among the mixture–monoculture comparisons, Bi + Cm had significantly greater aboveground biomass than Cm in 2024, whereas Bi + Ld had significantly greater aboveground biomass than Ld in both 2024 and 2025 (Figure 1a).

Vegetation cover was significantly affected by treatment and year, with no significant treatment × year interaction (Figure 1b; Table S1). All reseeding treatments significantly increased vegetation cover relative to the control in both years, with Bi + Ld showing the highest mean cover. In 2025, Bi + Ld also had significantly greater vegetation cover than the Ld monoculture (Figure 1b).

### 2.2. Effects of Reseeding Treatments on Plant Diversity Indices

**Figure 2:**
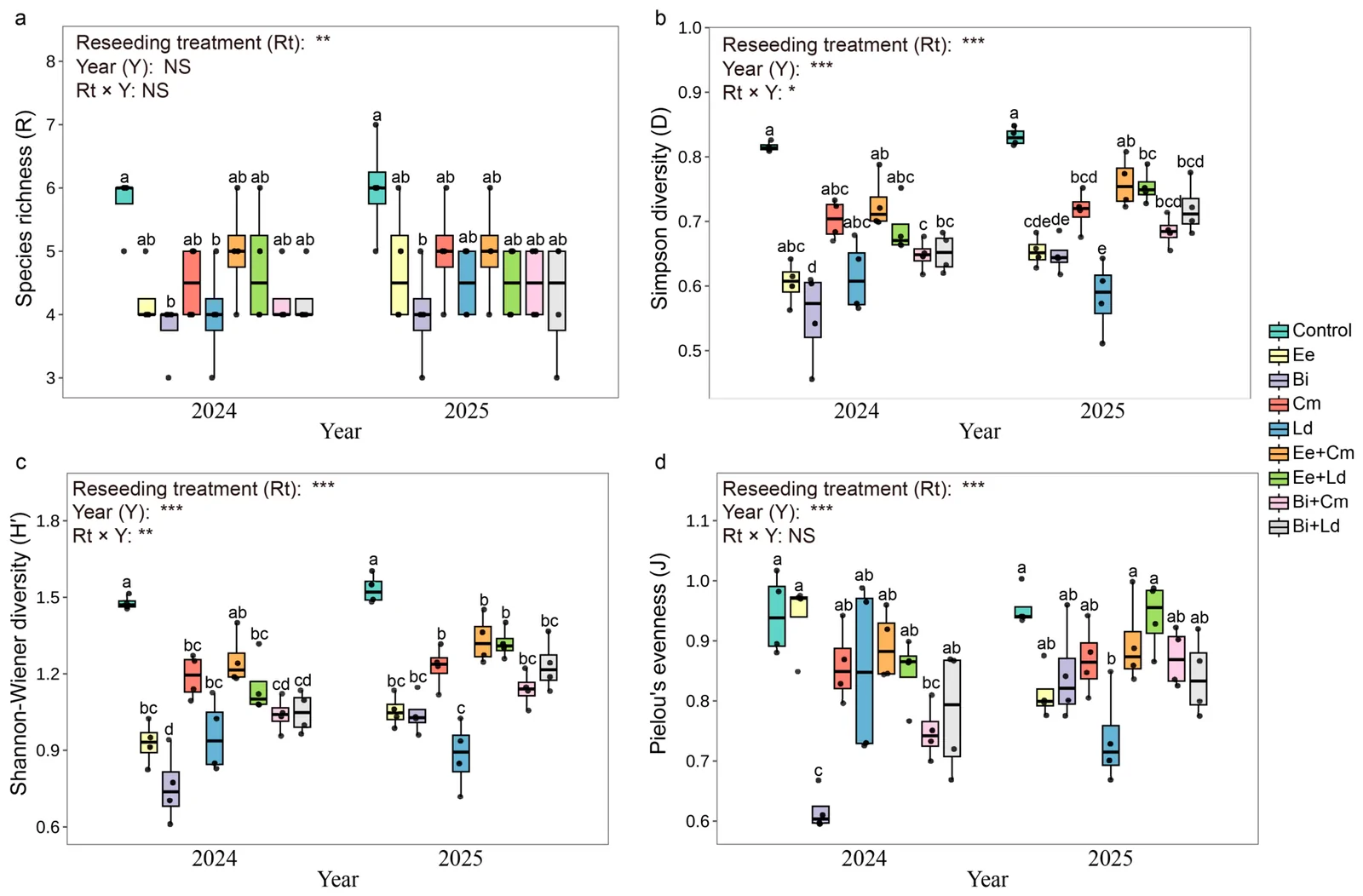
Effects of different reseeding treatments on (**a**) species richness, (**b**) the Simpson diversity index, (**c**) the Shannon–Wiener diversity index, and (**d**) Pielou’s evenness index in 2024 and 2025. Ee (*E. elongata*), Bi (*B. inermis*), Cm (*C. microphylla*), and Ld (*L. davurica*) represent four monocultures; Ee + Cm, Ee + Ld, Bi + Cm, and Bi + Ld represent four grass–legume mixtures. Data are presented as box plots with individual data points representing four independent experimental plots per treatment (*n* = 4). Boxes indicate the interquartile range, horizontal lines represent the median, and whiskers extend to the most extreme observations within 1.5 times the interquartile range. Different letters indicate significant differences among reseeding treatments within each year according to Tukey’s HSD test (*p* < 0.05). Treatments sharing at least one letter are not significantly different, whereas treatments with no letters in common differ significantly. Results of linear mixed-effects models for the effects of reseeding treatment (Rt), year (Y), and their interaction (Rt × Y) on species richness, the Simpson diversity index, the Shannon–Wiener diversity index, and Pielou’s evenness index are shown. *: *p* < 0.05; **: *p* < 0.01; ***: *p* < 0.001; NS: *p* > 0.05.

Reseeding treatment significantly affected species richness and Shannon–Wiener, Simpson, and Pielou’s evenness (Figure 2; Table S2). Year significantly affected the Simpson (Figure 2b), Shannon–Wiener (Figure 2c), and Pielou evenness (Figure 2d) indices but not species richness (Figure 2a). Significant treatment × year interactions were detected for the Simpson and Shannon–Wiener diversity indices, but not for species richness or Pielou’s evenness (Table S2).

Compared with the control, the Bi monoculture significantly reduced species richness in both years, whereas Ld reduced richness only in 2024. Species richness did not differ significantly from the control in any grass–legume mixture in either year (Figure 2a). Most reseeding treatments reduced the Shannon–Wiener and Simpson indices; however, Ee + Cm maintained both indices at levels comparable to the control in 2024 and 2025 (Figure 2b,c). Pielou evenness also showed species-specific responses: Bi and Bi + Cm significantly reduced evenness relative to the control in 2024, whereas Ld significantly reduced evenness in 2025 (Figure 2d). Among mixture–monoculture comparisons, Pielou’s evenness was significantly higher in Bi + Ld than in the Bi monoculture in 2024, whereas no other significant differences were detected between mixtures and their corresponding monocultures (Figure 2).

### 2.3. Effects of Reseeding Treatments on Soil Nutrients

**Figure 3:**
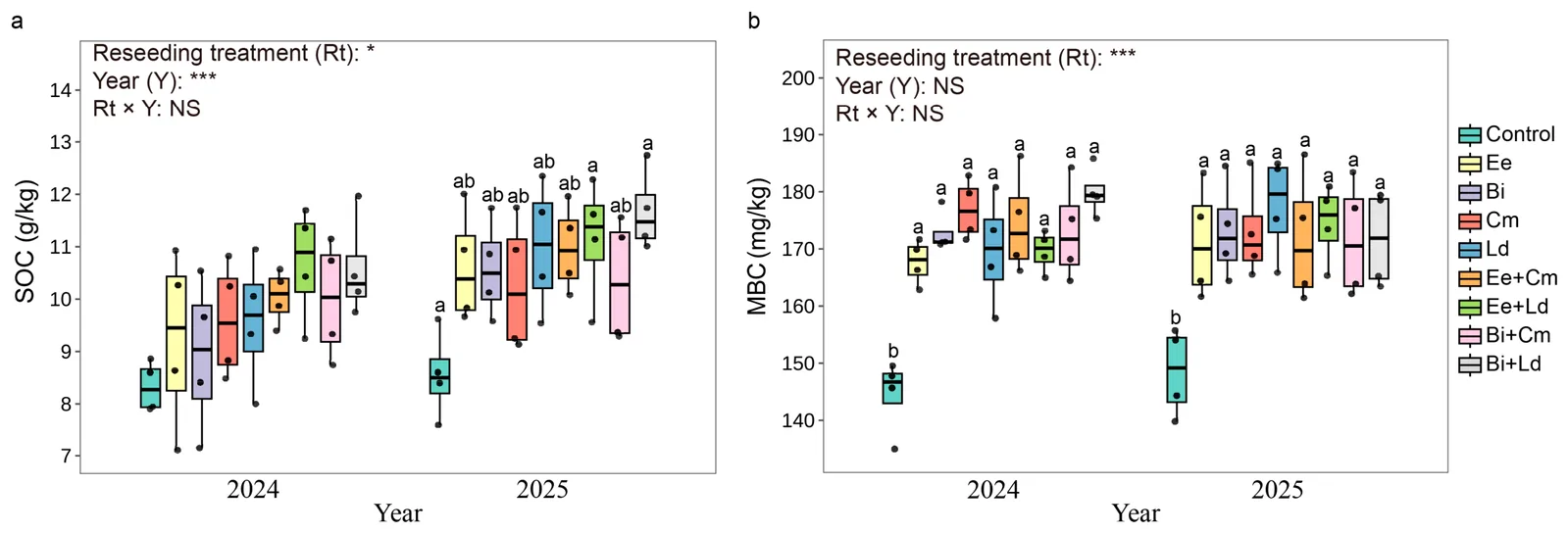
Effects of different reseeding treatments on (**a**) soil organic carbon (SOC) and (**b**) microbial biomass carbon (MBC) in 2024 and 2025. Ee (*E. elongata*), Bi (*B. inermis*), Cm (*C. microphylla*), and Ld (*L. davurica*) represent four monocultures; Ee + Cm, Ee + Ld, Bi + Cm, and Bi + Ld represent four grass–legume mixtures. Data are presented as box plots with individual data points representing four independent experimental plots per treatment (*n* = 4). Boxes indicate the interquartile range, horizontal lines represent the median, and whiskers extend to the most extreme observations within 1.5 times the interquartile range. Different letters indicate significant differences among reseeding treatments within each year according to Tukey’s HSD test (*p* < 0.05). Treatments sharing at least one letter are not significantly different, whereas treatments with no letters in common differ significantly. Results of linear mixed-effects models for the effects of reseeding treatment (Rt), year (Y), and their interaction (Rt × Y) on SOC and MBC are shown. *: *p* < 0.05; ***: *p* < 0.001; NS: *p* > 0.05.

Reseeding significantly affected soil organic carbon (SOC) and microbial biomass carbon (MBC), and year affected SOC but not MBC (Figure 3; Table S3). SOC did not differ from the control in 2024, but was significantly higher under Ee + Ld and Bi + Ld in 2025 (Figure 3a). MBC was significantly increased by all reseeding treatments in both years (Figure 3b), whereas soil pH remained unchanged across treatments (Figure S1).

**Figure 4:**
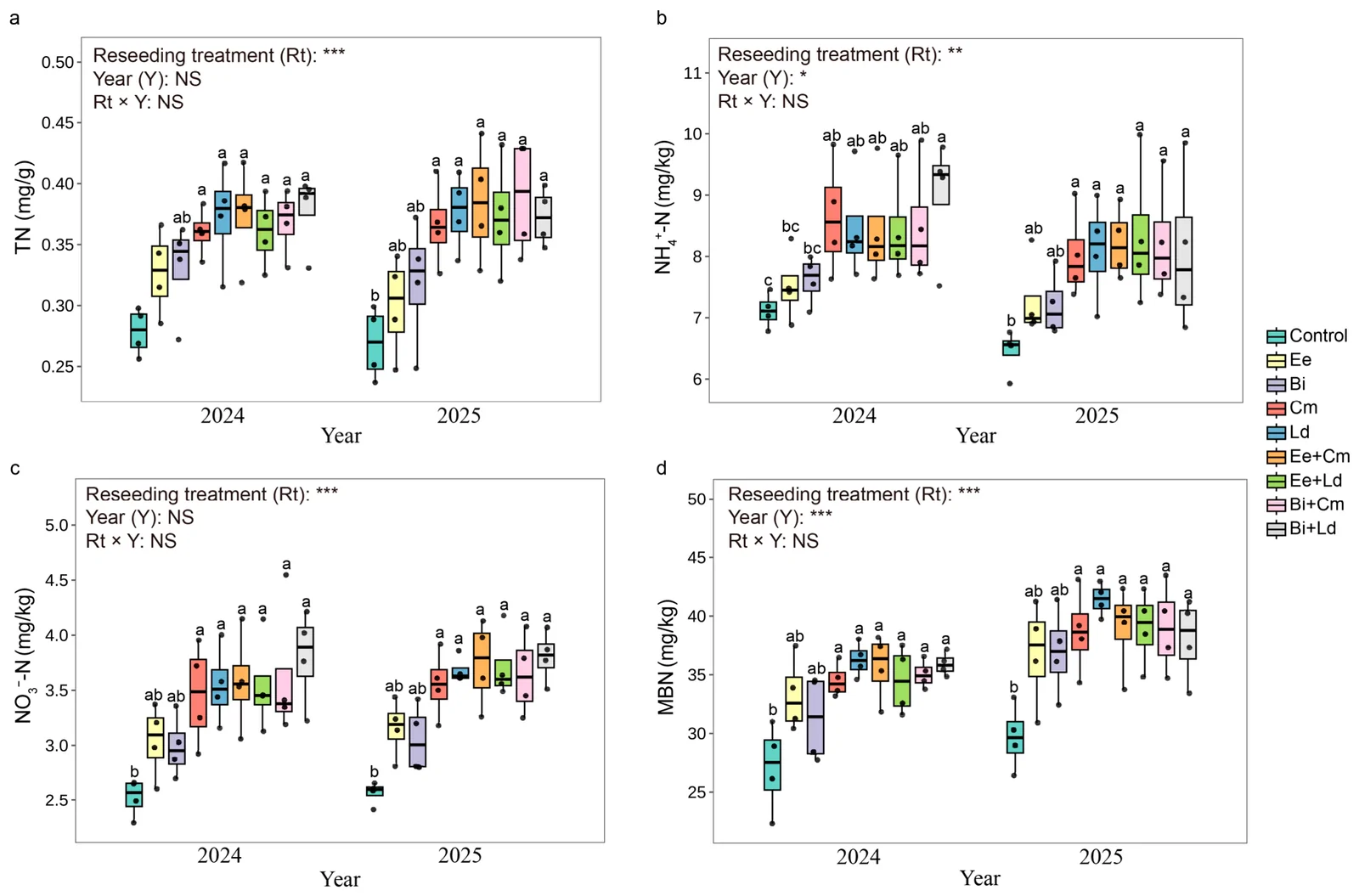
Effects of different reseeding treatments on (**a**) soil total nitrogen (TN), (**b**) soil ammonium nitrogen (NH_4_^+^-N), (**c**) soil nitrate nitrogen (NO_3_^−^-N), and (**d**) microbial biomass nitrogen (MBN) in 2024 and 2025. Ee (*E. elongata*), Bi (*B. inermis*), Cm (*C. microphylla*), and Ld (*L. davurica*) represent four monocultures; Ee + Cm, Ee + Ld, Bi + Cm, and Bi + Ld represent four grass–legume mixtures. Data are presented as box plots with individual data points representing four independent experimental plots per treatment (*n* = 4). Boxes indicate the interquartile range, horizontal lines represent the median, and whiskers extend to the most extreme observations within 1.5 times the interquartile range. Different letters indicate significant differences among reseeding treatments within each year according to Tukey’s HSD test (*p* < 0.05). Treatments sharing at least one letter are not significantly different, whereas treatments with no letters in common differ significantly. Results of linear mixed-effects models for the effects of reseeding treatment (Rt), year (Y), and their interaction (Rt × Y) on TN, NH_4_^+^-N, NO_3_^−^-N, and MBN are shown. *: *p* < 0.05; **: *p* < 0.01; ***: *p* < 0.001; NS: *p* > 0.05.

Reseeding significantly affected soil total nitrogen (TN), ammonium nitrogen (NH_4_^+^-N), nitrate nitrogen (NO_3_^−^-N), and microbial biomass nitrogen (MBN), while year significantly affected NH_4_^+^-N and MBN (Figure 4; Table S4). In both years, the Cm and Ld monocultures and all grass–legume mixtures significantly increased TN, NH_4_^+^-N, NO_3_^−^-N, and MBN relative to the control.

**Figure 5:**
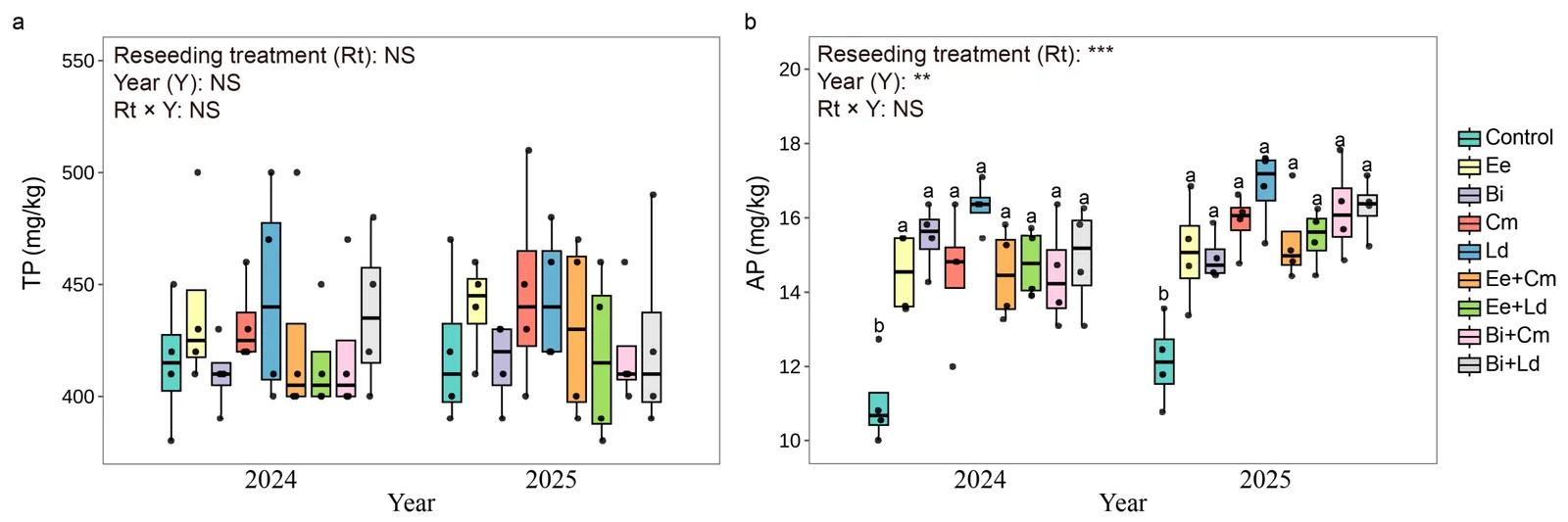
Effects of different reseeding treatments on (**a**) soil total phosphorus (TP) and (**b**) soil available phosphorus (AP) in 2024 and 2025. Ee (*E. elongata*), Bi (*B. inermis*), Cm (*C. microphylla*), and Ld (*L. davurica*) represent four monocultures; Ee + Cm, Ee + Ld, Bi + Cm, and Bi + Ld represent four grass–legume mixtures. Data are presented as box plots with individual data points representing four independent experimental plots per treatment (*n* = 4). Boxes indicate the interquartile range, horizontal lines represent the median, and whiskers extend to the most extreme observations within 1.5 times the interquartile range. Different letters indicate significant differences among reseeding treatments within each year according to Tukey’s HSD test (*p* < 0.05). Treatments sharing at least one letter are not significantly different, whereas treatments with no letters in common differ significantly. Results of linear mixed-effects models for the effects of reseeding treatment (Rt), year (Y), and their interaction (Rt × Y) on TP and AP are shown. **: *p* < 0.01; ***: *p* < 0.001; NS: *p* > 0.05.

Soil total phosphorus (TP) was unaffected by reseeding or year, whereas available phosphorus (AP) varied significantly with both factors (Figure 5; Table S5). All reseeding treatments significantly increased AP relative to the control in both years, while TP remained unchanged.

### 2.4. Relationships Among Vegetation Characteristics, Plant Diversity, and Soil Nutrients

**Figure 6:**
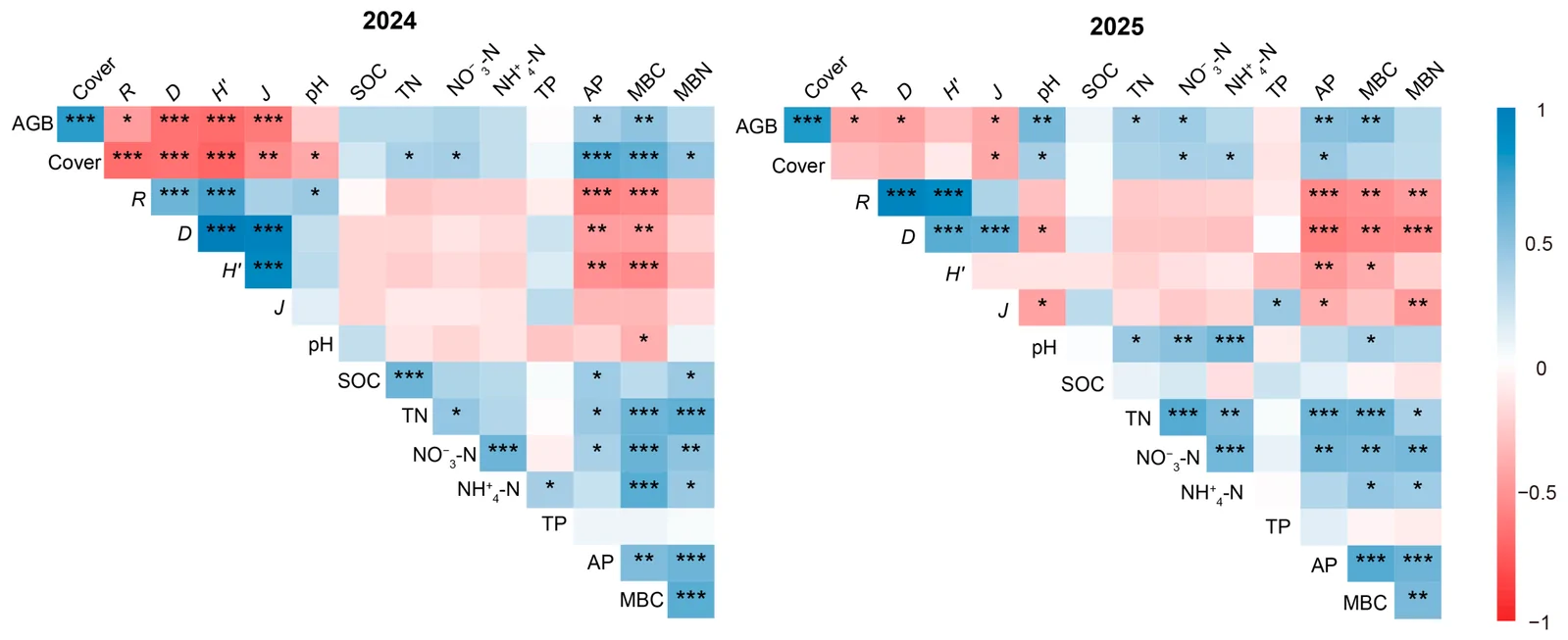
Block-adjusted Pearson correlation heatmap of vegetation and soil variables measured in 2024 and 2025. Asterisks denote significant correlations (* *p* < 0.05, ** *p* < 0.01, *** *p* < 0.001). AGB, aboveground biomass; *R*, species richness; *D*, Simpson diversity index; *H’*, Shannon–Wiener diversity index; *J*, Pielou evenness index; SOC, soil organic carbon; TN, total nitrogen; NO_3_^−^–N, nitrate nitrogen; NH_4_^+^–N, ammonium nitrogen; TP, total phosphorus; AP, available phosphorus; MBC, microbial biomass carbon; MBN, microbial biomass nitrogen.

Correlation patterns were broadly similar between 2024 and 2025, although the strength of several relationships varied between years (Figure 6). Aboveground biomass was positively correlated with vegetation cover but negatively correlated with species richness in both years. Biomass was also positively associated with AP and MBC in both years, with additional positive correlations with pH, TN, and NO_3_^−^-N in 2025.

Vegetation cover was negatively correlated with species richness, the Simpson diversity index, and Pielou’s evenness index. The Shannon–Wiener diversity index was also negatively associated with vegetation productivity, showing highly significant negative correlations with both aboveground biomass and vegetation cover in 2024 (Figure 6).

Vegetation cover was positively correlated with several soil nutrient variables. Plant diversity tended to be negatively associated with AP and microbial biomass, particularly in 2025. Positive correlations were also observed among soil nutrient availability and microbial biomass, including positive associations of AP with MBC and MBN and between MBC and MBN in both years (Figure 6).

## 3. Discussion

Reseeding promoted early vegetation recovery in a degraded semi-arid grassland, but the magnitude of the response depended on species combination. Vegetation cover increased under all reseeding treatments, whereas biomass gains were less consistent, showing that successful establishment did not always translate into proportional productivity gains. This overall pattern agrees with previous evidence that reseeding can improve vegetation cover and biomass in degraded grasslands [6,20].

Among treatments, the *B. inermis* and *L. davurica* mixture showed the highest mean cover and biomass in both years, while the other grass–legume mixtures did not consistently outperform monocultures. Grass–legume mixtures may benefit from complementary N acquisition because grasses depend mainly on soil mineral N, whereas legumes can also obtain N through symbiotic N_2_ fixation [11,12]. However, the contrasting performance of the four mixtures indicates that functional-group mixing alone does not guarantee greater productivity. Instead, restoration performance appears to depend strongly on the identities and interactions of the component species, consistent with species-specific mixture effects reported elsewhere [18,19]. The relatively high biomass and cover observed under *B. inermis* + *L. davurica* may partly reflect the performance of *B. inermis* itself; however, the contribution of interactions between the two species cannot be determined from our data. Other belowground processes remain possible explanations [21,22], but were not directly assessed in the current study.

The positive vegetation response was not accompanied by an increase in plant diversity. Most reseeding treatments reduced Shannon–Wiener and Simpson diversity, whereas species richness was generally maintained in the grass–legume mixtures. This divergence between productivity and diversity is consistent with reports that reseeding can increase biomass without increasing plant diversity [20], although the direction and magnitude of diversity responses can vary with degradation severity, restoration method, environmental conditions, and restoration duration [6,8].

Negative relationships between vegetation productivity and diversity suggest a potential trade-off during early restoration. In particular, Pielou’s evenness was negatively associated with aboveground biomass and vegetation cover, suggesting that higher productivity was associated with greater dominance by a subset of species. Rapid establishment of productive reseeded species can increase canopy development and competitive dominance, thereby reducing light and other resources available to less competitive resident species [23]. More generally, greater resource availability may favor fast-growing dominants and increase productivity while reducing diversity [17,24]. The positive associations of Pielou evenness with the Shannon–Wiener and Simpson indices further indicate that changes in abundance distribution contributed substantially to the observed diversity patterns.

Importantly, these responses differed among species combinations. The *E. elongata* + *C. microphylla* mixture maintained Shannon–Wiener and Simpson diversity at levels comparable to the unseeded control, whereas the *B. inermis* + *L. davurica* mixture produced the greatest vegetation cover and biomass. The combination that maximizes productivity may therefore differ from the one that best maintains plant diversity. The *B. inermis* + *L. davurica* mixture may be more suitable when rapid vegetation recovery is the primary objective, whereas *E. elongata* + *C. microphylla* may be a potential option when maintaining plant diversity is also a restoration objective.

Reseeding also improved soil nutrients, with particularly clear responses in legume-containing treatments. TN, NH_4_^+^-N, NO_3_^−^-N, and MBN were consistently higher in legume monocultures and grass–legume mixtures than in the control, suggesting an association between the presence of legumes and early soil N recovery. Although this pattern is consistent with the known capacity of legumes for symbiotic N_2_ fixation [10,11,12], N_2_ fixation and interspecific N transfer were not directly measured in this study. Therefore, these mechanisms cannot be confirmed as the causes of the observed soil N responses.

The increases in microbial biomass and available P across reseeded treatments likely reflect enhanced plant-derived C and nutrient inputs after vegetation establishment. Roots, rhizodeposits, and litter provide substrates for soil microorganisms [25], and the positive associations between microbial biomass and soil nutrient availability in both years suggest close coupling between vegetation recovery and belowground nutrient cycling. SOC concentrations were significantly higher than those in the contemporaneous unseeded control only in the *E. elongata* + *L. davurica* and *B. inermis* + *L. davurica* treatments in 2025. These results indicated differences in SOC concentrations among treatments at the same sampling time and should not be interpreted as changes relative to the pre-experimental SOC measurement, which was collected in a different season. Given the short experimental duration and potential seasonal variation in SOC, these differences do not demonstrate stable or long-term SOC accumulation. Recent plant-derived carbon inputs and residual fine roots or small organic materials may also have contributed to the observed differences. Longer-term monitoring with sampling at comparable seasonal stages, together with SOC fractionation, is needed to determine whether these differences persist over time [26,27].

This study captured only the first two years after reseeding and therefore represents an early stage of restoration. Because mixtures were standardized by seed mass rather than viable seed number, initial seed densities and species proportions differed among treatments. Thus, observed differences may reflect both species interactions and variation in initial propagule density, and should be interpreted accordingly. Although clear species-combination effects emerged across vegetation, diversity, and soil attributes, the underlying mechanisms cannot yet be fully resolved. In particular, N_2_ fixation, interspecific N transfer, root traits, and belowground chemical interactions were not directly measured. Therefore, longer-term studies integrating plant functional traits, N acquisition, and belowground processes will be needed to determine whether these species-specific differences persist as restored communities develop.

## 4. Materials and Methods

### 4.1. Study Site

The experiment was conducted in a degraded grassland in Chabugatu, Zhalute Banner, Tongliao, Inner Mongolia, China (44°14′ N, 120°55′ E), on the northwestern fringe of the Horqin Sandy Land (Figure S2a,b). Precipitation and daily temperature throughout the plant growing season in 2024 and 2025 are shown in Figure S2c. The dominant soils are chestnut and aeolian sandy soils. Long-term grazing and wind erosion have resulted in reduced vegetation cover, increased bare ground, and decreased soil nutrient availability. Grazing exclusion was implemented at the study site in 2023.

Before the experiment, the plant community was dominated by Poaceae species, particularly *Setaria viridis* and *Chloris virgata*, with *Tragus mongolorum*, *Artemisia frigida*, and *Convolvulus ammannii* as common associated species. Initial aboveground biomass and vegetation cover in the degraded grassland were approximately 30 g m^−2^ and 50%, respectively. Initial soil properties were determined in the 0–20 cm layer for characterization of the pre-experimental site conditions and were not used as a baseline in the statistical analysis of treatment effects. Soil pH was 7.27, and soil organic carbon (SOC), total nitrogen (TN), and total phosphorus (TP) concentrations were 18.03, 3.04, and 0.78 g kg^−1^, respectively. Ammonium nitrogen (NH_4_^+^-N), nitrate nitrogen (NO_3_^−^-N) and available phosphorus (AP) concentrations were 22.83, 8.27, and 11.51 mg kg^−1^, respectively.

### 4.2. Experimental Design and Reseeding Treatments

Four species were selected for reseeding based on preliminary vegetation surveys and their suitability for restoration of degraded sandy grasslands: two grasses, tall wheatgrass (*Elytrigia elongata*) and smooth brome (*Bromus inermis*), and two legumes, Chinese peashrub (*Caragana microphylla*) and Dahurian bushclover (*Lespedeza davurica*). Seeds were obtained from Beijing Best Grass Industry Co., Ltd. (Beijing, China), and all seed lots had germination rates of at least 75%.

A randomized complete block design was used, with four blocks established to account for spatial heterogeneity. Nine treatments were randomly assigned to plots within each block: an unseeded control (Control); four monocultures, *E. elongata* (Ee), *B. inermis* (Bi), *C. microphylla* (Cm), and *L. davurica* (Ld); and four grass–legume mixtures, Ee + Cm, Ee + Ld, Bi + Cm, and Bi + Ld. Each treatment was represented once in each block, resulting in four replicates per treatment and 36 experimental plots in total. Each plot measured 3 × 3 m, with a 2-m buffer between adjacent plots.

Monoculture treatments were sown at a rate of 40 g of a single species per plot. For mixture treatments, grass and legume seeds were combined at a 1:1 ratio by seed mass, with 20 g of each component species per plot. Thus, mixture composition was standardized on a seed-mass basis rather than by viable seed number. Because seed size and germination percentage differed among species, the resulting seed numbers and numerical species proportions were not identical among mixtures. Seed mass, 1000-seed weight, germination percentage, and the estimated seed and viable seed numbers for the 20-g component of each mixture are provided in Table S6. These estimates reflect seed characteristics and laboratory germination percentages rather than actual field emergence or subsequent survival.

Reseeding was conducted in late May 2024. Before sowing, all plots, including the unseeded controls, were shallow-tilled to approximately 10 cm in early April, and existing vegetation and surface litter were removed to improve seed–soil contact and reduce competition. Control plots received the same site preparation as the reseeded plots but were left unseeded. Seeds were manually broadcast in the reseeding treatments and lightly covered with 1–2 cm of soil using an iron rake. No fertilizer was applied during the experiment, and plant growth depended on natural precipitation.

### 4.3. Vegetation Survey and Aboveground Biomass Determination

Vegetation was surveyed in mid-August 2024 and 2025. Three 1 × 1 m quadrats were randomly located within each experimental plot as within-plot subsamples. Species composition, vegetation cover, plant height, and abundance were recorded in each quadrat. Total and species-specific cover were visually estimated, and abundance was recorded as the number of ramets or tillers. Plant height was measured for five randomly selected individuals of each species per quadrat; when fewer than five individuals were present, all individuals were measured.

Following the vegetation survey, all aboveground plant material within each quadrat was harvested at ground level, oven-dried at 65 °C to constant mass, and weighed to determine aboveground biomass. Measurements from the three quadrats were averaged within each plot before statistical analysis, with the plot treated as the experimental unit.

### 4.4. Soil Sampling and Physicochemical Analysis

Soil samples were collected in mid-September 2024 and 2025. Five soil cores (0–20 cm) were randomly collected from each plot as subsamples and combined into one composite sample representing that experimental plot. After removing visible roots and plant residues, subsamples were either air-dried for physicochemical analyses or stored at 4 °C and analyzed within one week for microbial biomass.

Soil pH was measured in a 1:2.5 (*w*/*v*) soil-to-water suspension. SOC and TN were determined by dry combustion, and TP by H_2_SO_4_–HClO_4_ digestion followed by colorimetry. NH_4_^+^-N and NO_3_^−^-N were extracted with 2 M KCl and quantified using a continuous-flow autoanalyzer. AP was extracted with 0.5 M NaHCO_3_ (pH 8.5) and determined colorimetrically.

Microbial biomass carbon (MBC) and nitrogen (MBN) were determined by chloroform fumigation–extraction [28,29]. MBC and MBN were calculated from the differences in extractable C and N between fumigated and unfumigated samples using extraction efficiency factors of k_EC_ = 0.45 and k_EN_ = 0.54, respectively. Fumigated and unfumigated soil subsamples were extracted with 0.5 M K_2_SO_4_, and extractable C and N were quantified using a total organic carbon analyzer and a continuous-flow autoanalyzer, respectively.

### 4.5. Data Analysis

Plant community diversity was quantified using species richness (*R*), the Simpson diversity index (*D*), the Shannon–Wiener diversity index (*H′*) and Pielou’s evenness index (*J*). These diversity indices were calculated based on species importance values, which were derived from the mean values of relative cover, relative height, and relative abundance. Calculation formulas are as follows:

Species richness:

*R* = *S* (1)

Simpson diversity index:

(2) $$D=1-\sum_{i=1}^{S}P_{i}^{2}$$

Shannon–Wiener diversity index:

(3) $$H^{\prime }=-\sum_{i=1}^{S}P_{i}\ln P_{i}$$

Pielou’s evenness index:

*J* = *H′*/ln *S* (4)

where *S* is the number of species and *P_i_* denotes the importance value of species *i*, calculated as the mean of relative cover, relative height, and relative abundance.

Linear mixed-effects models were used to test the effects of reseeding treatment, year, and their interaction on vegetation and soil variables. Reseeding treatment, year, and their interaction were included as fixed effects, whereas block and plot nested within block were included as random effects. This random-effects structure accounted for both spatial variation among blocks and repeated measurements of the same experimental plots in 2024 and 2025. When significant treatment effects were detected, pairwise comparisons among treatments were performed using Tukey-adjusted tests at *p* < 0.05.

Pearson correlations were calculated separately for 2024 and 2025 using plot-level residuals obtained after removing block effects with linear models to examine relationships among vegetation, diversity, and soil variables. Vegetation variables included aboveground biomass, vegetation cover, species richness, Shannon–Wiener diversity index, Simpson diversity index and Pielou’s evenness index. Soil variables included SOC, soil pH, TN, NH_4_^+^-N, NO_3_^−^-N, TP, AP, MBC, and MBN. All statistical analyses were performed using R software (version 4.4.2). Figures were generated using the ggplot2 package in R.

## 5. Conclusions

Our two-year field experiment demonstrated that reseeding promoted early vegetation and soil recovery in degraded semi-arid grassland, but responses depended strongly on species combination. The *B. inermis* + *L. davurica* mixture had the highest mean aboveground biomass and vegetation cover in both years, whereas the *E. elongata* + *C. microphylla* mixture maintained Shannon–Wiener and Simpson diversity indices at levels comparable to the unseeded control. Legume-containing treatments were associated with higher soil N availability and microbial biomass N than the control, while available P and microbial biomass C increased across all reseeding treatments. Importantly, grass–legume mixtures were not universally superior to monocultures, indicating that restoration outcomes depend on the identity of the species combined rather than functional-group mixing alone. These findings provide practical guidance for species selection in degraded grassland restoration, with *B. inermis* + *L. davurica* favoring vegetation recovery and *E. elongata* + *C. microphylla* offering a potential option for biodiversity maintenance. However, these recommendations are based on short-term responses and require further evaluation under longer-term and broader field conditions.

## Data Availability

All data supporting the findings of this research are available within the paper and Supplementary Materials.

## Supplementary Materials

The following supporting information can be downloaded at https://www.mdpi.com/article/10.3390/plants15203228/s1, Figure S1: Effects of different reseeding treatments on soil pH in 2024 and 2025; Figure S2: Location (a), photograph of the study area (b), and distribution of precipitation and air temperature (c) during the plant growing seasons of 2024 and 2025. Table S1: Results of linear mixed-effects models testing the effects of reseeding treatment, year, and their interaction on aboveground biomass and vegetation cover. Table S2: Results of linear mixed-effects models testing the effects of reseeding treatment, year, and their interaction on species richness, the Simpson diversity index, the Shannon–Wiener diversity index, and Pielou’s evenness index. Table S3: Results of linear mixed-effects models testing the effects of reseeding treatment, year, and their interaction on soil organic carbon (SOC) and microbial biomass carbon (MBC). Table S4: Results of linear mixed-effects models testing the effects of reseeding treatment, year, and their interaction on soil total nitrogen (TN), ammonium nitrogen (NH_4_^+^-N), nitrate nitrogen (NO_3_^−^-N), and microbial biomass nitrogen (MBN). Table S5: Results of linear mixed-effects models testing the effects of reseeding treatment, year, and their interaction on soil total phosphorus (TP) and available phosphorus (AP). Table S6: Seed mass, 1000-seed weight, germination percentage, estimated seed number, and estimated viable seed number of the four species used in grass–legume mixture treatments.
