# Supplementary material for: Species Combinations Shape Vegetation and Soil Nutrient Responses to Grass–Legume Reseeding in a Degraded Semi-Arid Grassland
Source: Plants (Basel). 2026 Oct 21;15(20):3228. doi: 10.3390/plants15203228 (PMC13708903; doi:10.3390/plants15203228)
Supplement: Supplementary file 1 [file plants-15-03228-s001.zip › plants-4608154-supplementary.pdf]

Supplementary materials

Species combinations shape vegetation and soil nutrient responses to grass–legume reseeding in a degraded semi-arid grassland

You Xu [1], Guang Liu [1], Tian-Sheng Fu [2], Gui-Hong Fu [3], Jin-Xin Zhang [1,*] and Chui-Hua Kong [4,*]

This supplementary material was prepared to add the readers more details, for which there was not enough space in the main manuscript, about:

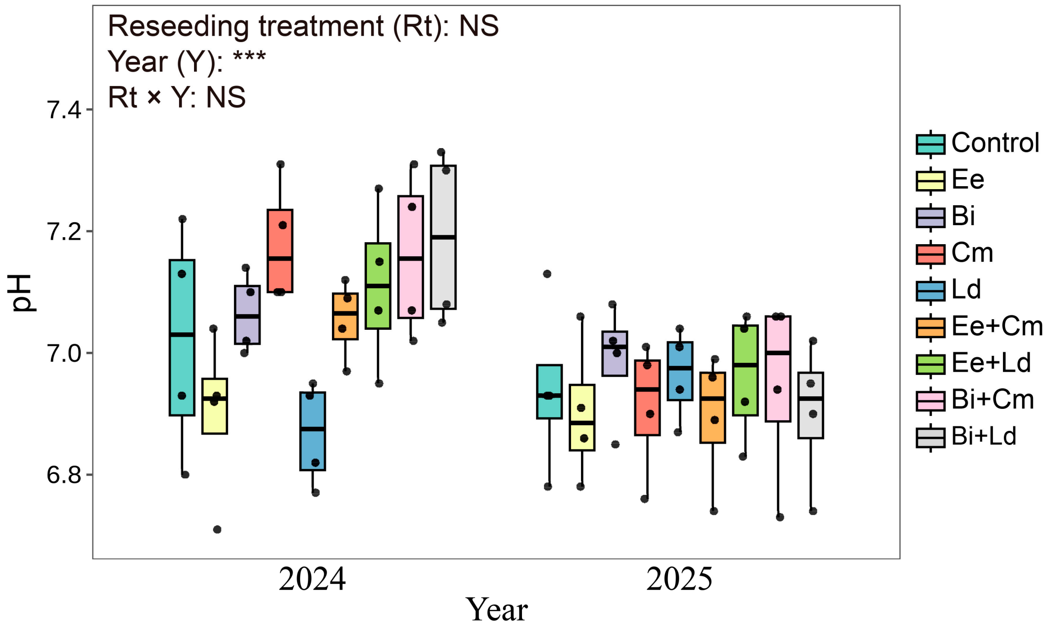

Figure S1. Effects of different reseeding treatments on soil pH in 2024 and 2025. Ee (*E. elongata*), Bi (*B. inermis*), Cm (*C. microphylla*), Ld (*L. davurica*) represent four monocultures; Ee+Cm, Ee+Ld, Bi+Cm, and Bi+Ld represent four grass–legume mixtures. Data are presented as box plots with individual data points representing four independent experimental plots per treatment (n = 4). Boxes indicate the interquartile range, horizontal lines represent the median, and whiskers extend to the most extreme observations within 1.5 times the interquartile range. Different letters indicate significant differences among reseeding treatments within each year according to Tukey's HSD test ($p < 0.05$). Results of linear mixed-effects models for the effects of reseeding treatment (Rt), year (Y), and their interaction (Rt × Y) on soil pH are shown. *: $p < 0.05$; **: $p < 0.01$; ***: $p < 0.001$; NS: $p > 0.05$.

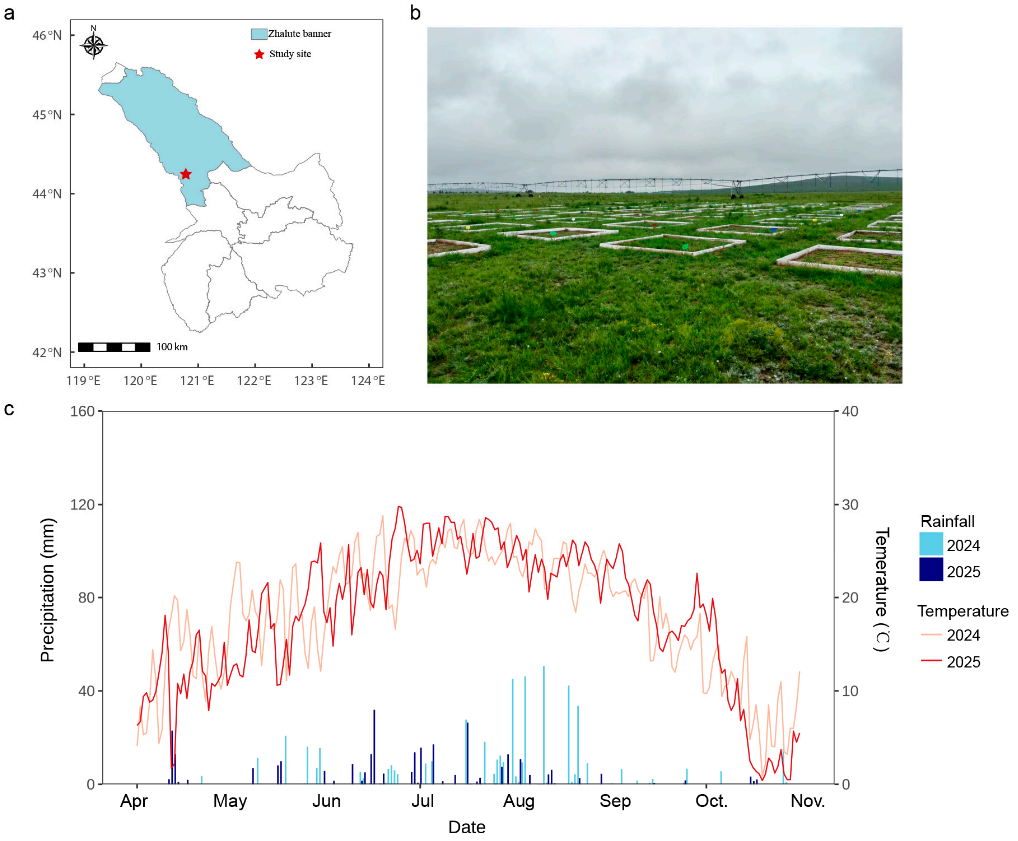

Figure S2. Location (a), photograph of the study area (b), and distribution of precipitation and air temperature (c) during the plant growing seasons of 2024 and 2025.

Table S1. Results of linear mixed-effects models testing the effects of reseeding treatment, year, and their interaction on aboveground biomass and vegetation cover. Effects are reseeding treatment (*E. elongata*, *B. inermis*, *C. microphylla*, *L. davurica*, *E. elongata* + *C. microphylla*, *E. elongata* + *L. davurica*, *B. inermis* + *C. microphylla*, and *B. inermis* + *L. davurica*) and year (2024 vs. 2025). Significance of effects was determined by Satterthwaite's approximate F-test.

**Effects**	**NumDF**	**DenDF**	**Aboveground biomass**		**Cover**	
			F	***P***	***F***	***P***
Reseeding treatment (Rt)	8	24	18.038	<0.001	17.139	<0.001
Year (Y)	1	27	48.608	<0.001	63.157	<0.001
Rt × Y	8	27	1.140	0.369	1.164	0.355
(random factor) Block/Plot						

Table S2. Results of linear mixed-effects models testing the effects of reseeding treatment, year, and their interaction on species richness, the Simpson diversity index, the Shannon–Wiener diversity index, and Pielou's evenness index. Effects are reseeding treatment (*E. elongata, B. inermis, C. microphylla, L. davurica, E. elongata* + *C. microphylla, E. elongata* + *L. davurica, B. inermis* + *C. microphylla*, and *B. inermis* + *L. davurica*) and year (2024 vs. 2025). Significance of effects was determined by Satterthwaite's approximate F-test.

**Effects**	**NumDF**	**DenDF**	**Species richness**		**Simpson diversity index**		**Shannon–Wiener diversity index**		**Pielou's evenness index**	
			F	***P***	***F***	***P***	***F***	***P***	***F***	***P***
Reseeding treatment (Rt)	8	24	4.830	0.001	28.527	<0.001	29.158	<0.001	7.211	<0.001
Year (Y)	1	27	1.846	0.185	24.739	<0.001	36.913	<0.001	5.150	<0.001
Rt×Y	8	27	0.288	0.963	2.561	0.032	3.740	0.004	3.608	0.068
(random factor) Block/Plot										

.

Table S3. Results of linear mixed-effects models testing the effects of reseeding treatment, year, and their interaction on soil organic carbon (SOC) and microbial biomass carbon (MBC). Effects are reseeding treatment (*E. elongata*, *B. inermis*, *C. microphylla*, *L. davurica*, *E. elongata* + *C. microphylla*, *E. elongata* + *L. davurica*, *B. inermis* + *C. microphylla*, and *B. inermis* + *L. davurica*) and year (2024 vs. 2025). Significance of effects was determined by Satterthwaite's approximate F-test.

**Effects**	**NumDF**	**DenDF**	**SOC**		**MBC**	
			F	***P***	***F***	***P***
Reseeding treatment (Rt)	8	24	2.797	0.024	10.856	<0.001
Year (Y)	1	27	21.221	<0.001	0.071	0.791
Rt × Y	8	27	0.733	0.661	0.849	0.568
(random factor) Block/Plot						

Table S4. Results of linear mixed-effects models testing the effects of reseeding treatment, year, and their interaction on soil total nitrogen (TN), ammonium nitrogen (NH_4^+-N), nitrate nitrogen (NO_3^--N), and microbial biomass nitrogen (MBN). Effects are reseeding treatment (*E. elongata*, *B. inermis*, *C. microphylla*, *L. davurica*, *E. elongata* + *C. microphylla*, *E. elongata* + *L. davurica*, *B. inermis* + *C. microphylla*, and *B. inermis* + *L. davurica*) and year (2024 vs. 2025). Significance of effects was determined by Satterthwaite's approximate F-test.

Effects	NumDF	DenDF	TN		NH_4^+-N		NO_3^--N		MBN	
			F	***P***	***F***	***P***	***F***	***P***	***F***	***P***
Reseeding treatment (Rt)	8	24	8.210	<0.001	3.437	0.009	8.476	<0.001	7.953	<0.001
Year (Y)	1	27	0.001	0.983	6.740	0.015	1.630	0.212	29.197	<0.001
Rt×Y	8	27	0.313	0.954	0.302	0.959	0.088	0.999	0.278	0.967
(random factor) Block/Plot										

Table S5. Results of linear mixed-effects models testing the effects of reseeding treatment, year, and their interaction on soil total phosphorus (TP) and available phosphorus (AP). Effects are reseeding treatment (*E. elongata, B. inermis, C. microphylla, L. davurica, E. elongata* + *C. microphylla, E. elongata* + *L. davurica, B. inermis* + *C. microphylla*, and *B. inermis* + *L. davurica*) and year (2024 vs. 2025). Significance of effects was determined by Satterthwaite's approximate F-test.

Effects	NumDF	DenDF	TP		AP	
			F	*P*	*F*	*P*
Reseeding treatment (Rt)	8	24	0.550	0.806	11.303	<0.001
Year (Y)	1	27	0.194	0.663	10.118	0.003
Rt × Y	8	27	0.292	0.962	0.676	0.707
(random factor) Block/Plot						

Table S6. Seed mass, 1000-seed weight, germination percentage, estimated seed number, and estimated viable seed number of the four species used in grass–legume mixture treatments.

Species	Seed mass used in mixture (g)	1000-seed weight (g)	Germination (%)	Estimated seed number per plot	Estimated viable seed number per plot
E. elongata	20	5.1	80	3922	3137
B. inermis	20	3.7	75	5405	4054
C. microphylla	20	26.2	85	763	649
L. davurica	20	4.6	75	4348	3261

Note: Estimated seed number was calculated from seed mass and 1000-seed weight, and estimated viable seed number was calculated by multiplying the estimated seed number by the germination percentage. Values were rounded to the nearest whole number.
